# Vaccinology Education of Nurses and the Current Immunoprophylaxis Recommendations for Children with Juvenile Idiopathic Arthritis

**DOI:** 10.3390/jcm9113736

**Published:** 2020-11-20

**Authors:** Anna Bednarek, Robert Klepacz

**Affiliations:** 1 Department of Paediatric Nursing, Faculty of Health Sciences, Medical University of Lublin, 20059 Lublin, Poland; 2 Department of Clinical Pathomorphology, Medical University of Lublin, 20059 Lublin, Poland; robertklepacz@umlub.pl

**Keywords:** juvenile idiopathic arthritis, preventive vaccinations, vaccine administration recommendations, vaccinology education

## Abstract

Introduction: The immunosuppressive effect of the disease and the applied treatment in children with juvenile idiopathic arthritis increases the risk of infections. It is therefore essential that vaccinations be properly implemented and that a proper serological response is provoked after the vaccination. A competent nurse acting in compliance with the current recommendations constitutes one of the safety pillars of immunization of pediatric patients with juvenile idiopathic arthritis. Aim: To discuss evidence-based recommendations for immunization of pediatric patients with juvenile idiopathic arthritis in the context of nursing vaccination practice and vaccinology education. Material and Methods: A systematic review of the literature presenting evidence-based recommendations of the European League Against Rheumatism (EULAR) expert group on immunization of children with juvenile idiopathic arthritis. Compilation of source data selected subjectively by the authors in a standard literature search of Medline, Cochrane and Scopus databases, including both recommendations for immunization of children with juvenile idiopathic arthritis and the tasks to be performed by nurses in the course of vaccine administration. As part of the standard literature review of Medline, Cochrane and Scopus databases, including both recommendations for immunization of children with juvenile idiopathic arthritis and the tasks to be performed by nurses in the course of vaccine administration. Results: Most vaccines are immunogenic and safe for patients with juvenile idiopathic arthritis. The use of attenuated vaccines in patients receiving long-term immunosuppressive treatment should be considered with particular caution. Education and further training of nurses should take into account the recommendations and principles of immunization regarding children with juvenile idiopathic arthritis. Nurses should present the current knowledge of active immunoprophylaxis in such a way as to encourage parents/guardians to vaccinate their children in accordance with the national guidelines. Conclusion: The recommendations of the European League Against Rheumatism place special emphasis on the use of active immunoprophylaxis in the form of vaccination in children with juvenile idiopathic arthritis. The immunization schedule must be adjusted to the applied JIA treatment regimen. Such a stance on this matter is highly important as treatment regimens increasingly include biological drugs. Correctly performed by a nurse, a vaccination procedure is an important determinant of the desired immunoprophylactic results and minimizes the risk of adverse events following immunization. The priority for a nurse who provides active immunoprophylaxis should be to systematically broaden her training in immunization of chronically ill children, including juvenile idiopathic arthritis.

## 1. Introduction

Juvenile idiopathic arthritis (JIA), also known as juvenile rheumatoid arthritis, juvenile chronic arthritis or Still’s disease, is the most common form of arthritis in children. Its etiology is unknown. Generally, it has been indicated to have an autoimmune background [1]. The JIA starts before the age of 16 with a flare lasting for at least 6 weeks. The disease is accompanied by changes in the locomotor system and extra-articular manifestations. The consequence of the inflammatory process is joint destruction leading to deformities and mobility disorders. Rapid muscle atrophy leads to joint contractures, which in turn may lead to disability [2]. However, thanks to the progress that has been made in the treatment of this disease in recent years and the introduction of modern therapies, such complications are nowadays rare [1]. The global annual incidence rate of JIA is estimated to be 8 per 100,000 children, and the prevalence ranges between 15 and 150 per 100,000 children (in Europe between 3.8 and 400 per 100,000), depending on the geographical region and the survey methodology [3,4].

JIA is a chronic disease with periods of exacerbation and periods of remission. Pharmacological treatment involves aggressive combination therapy. As of date, disease-modifying drugs and immunosuppressive drugs are used. Biological therapy is a therapeutic alternative in patients with high disease activity in the absence of response to DMARD (mostly MTX). Both the immunological background and the therapy regimens may constitute important factors affecting active immunoprophylaxis in children with JIA [1,2,5].

Administration of vaccinations to healthy children, as well as to children with various chronic diseases, after medical examination and a pre-vaccination screening conducted by a doctor, is mainly performed by nurses. Awareness of the current recommendations for immunization of children with JIA is a key element of the proper implementation of the national immunization program (NIP). It also provides nurses with comprehensive knowledge, allowing them to talk constructively with parents/guardians and/or children about the administered vaccines [6,7].

## 2. Aim

The aim of this paper is to discuss the current recommendations of EULAR/PReS experts [EULAR (European League Against Rheumatism)/PReS (Pediatric Rheumatology European Society] for immunization of children with JIA, which are based on the available scientific data. Clear and unambiguous criteria are important for the effective implementation of routine immunization. Therefore, in order to ensure proper implementation of NIP in children with chronic diseases, including JIA, it is necessary to educate nurses in vaccinology.

## 3. Materials and Methods

A systematic review of literature presenting recommendations of EULAR/PReS experts for immunization of children with juvenile idiopathic arthritis. The recommendations were developed in line with standard operating procedures of the European League Against Rheumatism. Compilation of source data selected subjectively by the Authors in a standard literature search of Medline, Cochrane and Scopus databases, including both recommendations for immunization of children with juvenile idiopathic arthritis and the tasks to be performed by nurses in the course of vaccine administration.

### Process of Making Recommendations

Evidence-based recommendations for vaccination of pediatric patients with JIA were developed following the EULAR standardized procedure for guideline development.

The EULAR task force was instituted, and it was composed of eight pediatric rheumatologists/immunologists (I.K.-P., A.F., K.M., A.R., M.A., G.S.P., M.B., N.M.W.), one adult rheumatologist/immunologist (M.D.), one expert in immunization (R.B.), one expert in public health and infectious disease control (F.vdK.), one epidemiologist (K.M.) and two physicians/Ph.D. students in charge of the systematic literature review (M.W.H., L.M.OdB.).

First, the expert committee defined the search terms for the systematic literature review, which was performed by searching the Medline database in December 2009, Medline and Embase in November 2010 and the abstracts from EULAR and American College of Rheumatology (ACR) meetings of 2008/9. Exclusion criteria were: non-rheumatic autoimmune diseases, malignancies, immunodeficiencies, transplantations, atopic diseases, animal studies, infections rather than vaccinations, vaccine development, phase I–III clinical trials, in vitro studies and non-English papers. Papers concerning the potential role of vaccinations in inducing rheumatic diseases were also excluded because the presented recommendations focus on the effect of vaccination on patients with pre-existing disease.

The results of studies on adult patients with rheumatic diseases were extrapolated to juvenile patients. The results of the critical evaluation of the publications were debated, and subsequently, recommendations were formulated [6,8,9,10,11,12,13,14].

The expert committee performed a critical evaluation of 60 articles on vaccinations and immunosuppressive drugs and 147 articles on vaccinations and autoimmune rheumatic diseases. Eventually, 107 articles and 8 abstracts were selected and used. They contained meta-analyses of randomized trials (Level IA), randomized trials (Level IB), prospective controlled interventional trials without randomization (Level II), observational studies, including case–control studies, cross-sectional studies and case series (Level III) and reports and opinions of expert groups and/or clinical experiments of respected authorities (Level IV). Most papers concerned vaccinations against seasonal influenza and pneumococcal infections. Twenty-six of the reviewed studies were conducted specifically in pediatric patients, and the remaining papers contained data on adults with rheumatoid arthritis or systemic lupus erythematosus. Fifteen recommendations were developed with an overall agreement from 7.9 to 9.8. Delphi voting method was applied to determine the level of agreement between the task force members. If the level of agreement was below 7.5, the recommendation was not incorporated in the guidelines. All studies concerned the efficacy of vaccination based on serological response. The analyzed scientific data were classified according to the quality of scientific content (Levels I–IV) and strength of recommendations (Grades A–D), where Grade A and B signify strong recommendations, evidence level I and II; and Grades C and D signify weak recommendations, evidence level III and IV. The overall agreement level among the members of the expert committee with regard to the recommendations was 91.7%. However, due to the lack of information on many vaccinations, diseases and immunosuppressive drugs, the strength of most recommendations is graded C or D.

## 4. Results

Thirteen out of 15 recommendations of the EULAR expert group for vaccination of children with rheumatic diseases are applicable to children with JIA. Among these, we can distinguish recommendations that address the administration of vaccinations with respect to immunosuppressive drugs (illustrated in Table 1) and recommendations for the administration of inactivated and attenuated vaccinations (illustrated in Table 2 and Table 3, respectively). The recommendations refer to the local, national immunization programs (NIPs), which take into account the local health policies, including epidemiology and organizational aspects. National Immunization Programs often show significant differences between countries [7,15,16,17].

### 4.1. Recommendations for the Administration of Vaccines in Children with JIA

In view of an increased risk of infections in children with JIA, resulting primarily from the treatment used, the administration of vaccines against diseases that can be prevented by vaccination may be important for children with this disease. At the same time, it should be remembered that some of these children, for various reasons, may not have had a complete vaccination schedule implemented in their early childhood [16].

A number of studies have shown that inactivated vaccines used in children with JIA, who are treated with GCs, DMARDs or TNF-α antagonists do not exacerbate the course of the disease. Furthermore, no evidence was found for severe vaccine adverse events (VAE) to occur more frequently than in the healthy population [7,18,19]. This was demonstrated for hepatitis B vaccination [20] and influenza and PPSV-23 vaccinations administered during the treatment with GCs and methotrexate [21,22]. Data on other DMARDs with respect to the occurrence of severe VEs are limited [23,24]. In patients receiving rituximab, there was no change in the disease activity after influenza vaccination. VAE incidence after influenza, PPSV–23 [25], and tetanus (TT) vaccinations was comparable to that in patients who were not receiving rituximab [26,27]. During low-dose administration of GCs (<0.5–2.0 mg/kg BW/24 h), a good serological response to vaccination was obtained [15,21]. However, TT and influenza vaccination administered one month after rituximab treatment resulted in a weakened immune response [21,28]. When these vaccines were administered 6–10 months after rituximab treatment, the responses ranged from sufficient [26,29] to weakened [30]. Therefore, the EULAR expert group recommends the administration of specific tetanus immunoglobulin in patients who have indications for tetanus prophylaxis in contaminated wound management and who have been taking rituximab in the past six months. Post-vaccination response to PPSV–23 vaccine administered 6–7 months after rituximab treatment was also weakened, so it is proposed to consider a conjugate vaccine (PCV), which may be more immunogenic in immunosuppressed patients [31]. In the case of new biologicals, there is not enough scientific data to allow to formulate recommendations on how to evaluate specific post-vaccination antibodies [25]. These observations suggest that (whenever possible) vaccinations should be administered before starting treatment with rituximab or biological drugs.

According to the manufacturers’ recommendations, attenuated vaccines should not be administered to immunosuppressed patients due to the risk of infection by microorganisms contained in the vaccine preparation. For this reason, the EULAR expert group recommends delaying the administration of attenuated vaccines in patients receiving high-dose GCs, DMARDs or biological drugs until more data are available [1,7,17,24].

The MMR and VZV vaccinations are safe and effective for patients who are not on immunosuppressive drugs. Immunogenicity of MMR booster dose was confirmed as satisfactory in the studies conducted in children with JIA, irrespective of long-term methotrexate treatment [32].

As of date, the safety of the tuberculosis vaccine (BCG) has not been studied in the population of children with JIA. At the same time, the risk of tuberculosis is increased in patients treated with immunosuppressive drugs, especially TNF-α antagonists [33]. BCG vaccine should, therefore, be administered before the start of immunosuppressive therapy [34]. According to general recommendations, immunosuppressed children should be vaccinated against influenza every year [35]. Studies have shown that seasonal influenza vaccination was safe and immunogenic in children with JIA and reduced the frequency of viral and bacterial infections [36]. In addition, most studies have confirmed the safety and immunogenicity of diphtheria, Hib, hepatitis A and hepatitis B vaccines [15,20] and meningococcal conjugate vaccines [37,38].

According to the recommendations, administration of inactivated vaccines during therapy with low doses of GCs (2.5–40 mg/24 h), methotrexate (7–25 mg/week) or other DMARDs (e.g., azathioprine) or biological drugs appears to be safe. It is reasonable to refrain from administering attenuated vaccines, especially during the primary vaccination (first doses of the vaccine), in patients undergoing treatment with high-dose immunosuppressive or biological drugs. Usually, primary vaccinations (during infancy and early childhood) will have already been completed prior to the onset of the disease. However, there are no definitive contraindications for administering booster doses of attenuated vaccines [39,40].

The effect of immunosuppressive drugs and the disease itself on the persistence of protective concentration of specific antibodies in the serum has still not been determined. In addition, further research is required to assess the administration of vaccinations in the context of the incidence of infectious diseases and their complications in the course of JIA [5,6,7].

In 2019, the EULAR expert group updated the guidelines for the use of vaccinations in autoimmune inflammatory rheumatic diseases in adults. Among six of their universal recommendations for vaccination in patients with rheumatic diseases, there are recommendations that deserve special attention as they are also applicable in children. These include a regular assessment (once a year) of the immunization level and indications for subsequent vaccinations. The assessment should be performed by a team of doctors working in a specialist rheumatological institution, which is taking care of the patient. In addition, this team shall also be responsible for discussing an individual vaccination plan with the patient and/or parent/guardian, enabling them to participate in making an informed decision. Vaccinations should be administered as a result of cooperation between a general practitioner and a rheumatologist, with the participation of the patient and/or parent/guardian and the vaccination nurse [41].

### 4.2. Tasks for Nurses Administering Vaccinations in Children with JIA

Adequate implementation of immunization shall be a priority for the team responsible for the administration of vaccinations, especially in children with chronic diseases, including JIA. Any negligence in this area may result in the occurrence of VEs and their negative consequences for the patient [42,43].

In most countries, it is the nurses who are responsible for administering vaccinations. They administer vaccinations to healthy children as well as to children in special risk groups and children with diagnosed chronic diseases. Table 4 lists the most important elements of a vaccination procedure in children with JIA and the tasks to be performed by an immunization nurse.

It is the duty of the nurse who administers a vaccine to a patient with JIA to pay attention to several important issues during the implementation of vaccination procedure. She is obliged to carefully check the patient’s identity, confirm the type of vaccination to be administered with the patient and/or the parent/guardian, and ensure that their expectations are consistent with the indications for the vaccination. It is then important to inquire about any adverse events or exacerbation of the underlying disease that may have occurred after the last vaccination [44]. It is advisable to make sure that a few days before the scheduled vaccination of a child with JIA, no one in the child’s immediate environment, e.g., younger siblings, received an attenuated vaccine (e.g., against rotaviruses, varicella) because there is a potential risk of transmitting the virus onto the child with JIA. In such a case, the planned vaccination should be postponed, and the child and/or the parent/guardian should be informed that it is important to keep the rules of personal hygiene and avoid contact with the vaccinated household member for a week [45].

Next, it is necessary to check that there are no contraindications to vaccination. The nurse is obliged to carefully verify the written recommendations of the physician who qualified the patient for the vaccination. Vaccinations should be administered during remission of the underlying disease. The standard check also involves verification of the vaccine preparation (expiry date, route of administration and dose, which may sometimes be customized for patents treated for JIA). While administering a vaccination, it is important to follow the manufacturer’s recommendations and carefully choose the anatomical site and the technique of administration. Furthermore, the most effective method of soothing the pain that may accompany the vaccination should be recommended. Before administering a vaccination, it is important to ensure that the anatomical site recommended by the manufacturer can be used in a child with JIA (e.g., there are no skin lesions, swelling, redness). If this is the case, the nurse should consult the doctor and agree on an alternative injection site. It should also be noted that the proper technique of administration allows obtaining the desired immunoprophylactic results [46,47].

It is also vital that the vaccination nurse provide reliable information about potential adverse events that may follow immunization and maintain detailed vaccination documentation. After vaccination, the patient should remain under observation at the medical facility for at least 30 min. People cohabitating with a child who often undergoes immunosuppression should be encouraged to receive immunization in compliance with national guidelines in order to reduce the risk of exposing the child to infectious diseases [43].

As part of her professional tasks in the field of health promotion and disease prevention, it is also the nurse’s duty to inform in an accessible way the child and the parent/guardian about the benefits and risks of vaccinations. Priority should be given to providing the patients with informational materials based on reliable scientific facts that can be trusted. The immunoprophylactic knowledge of nurses working in vaccination surgeries should, therefore, be regularly supplemented, in particular, by up-to-date data on vaccination options for children with chronic diseases [48,49].

## 5. Conclusions

Immunization in children with JIA is important and should be implemented. The immunization schedule needs to be adjusted to the child’s health status, the phase of the disease and the type of vaccination, with respect to the national immunization program. The recommendations of the EULAR expert group put particular emphasis on the use of active immunoprophylaxis in the form of vaccination in children with juvenile idiopathic arthritis. The immunization schedule must be adjusted to the applied JIA treatment regimen. Such a stance on this matter is highly important as treatment regimens increasingly include biological drugs.

Vaccinations may be less effective in patients with JIA, but the safety profile is similar to that of healthy individuals. Correctly performed by a nurse, a vaccination procedure is an important determinant of the desired immunoprophylactic results and minimizes the risk of VAE.

### Key Issues Raised by the Article

- The course and severity of juvenile idiopathic arthritis constitute important factors in determining the immunization schedule;
- The recommendations formulated by the EULAR expert group address vaccination issues in children with juvenile idiopathic arthritis (JIA);
- A competent vaccination nurse has the necessary knowledge about vaccinations and contributes to their proper implementation in children with juvenile idiopathic arthritis.

## Figures and Tables

**Table 1:** Recommendations of the European League Against Rheumatism (EULAR) expert group for immunization of children with Juvenile idiopathic arthritis (JIA) during immunosuppressive treatment.

Recommendation Number	Treatment Regimen and Recommendation	Grade of Recommendation	Delphi Score * Mean (SD)
1.	GCs, DMARDs and/or TNF-α antagonists: children may be administered inactivated vaccines according to the local NIP	C	9.8 (0.4)
2.	High-dose GCs ^1^ or rituximab: it is recommended to determine the concentration of specific antibodies after vaccination. Evaluation of antibodies may also be considered in patients on anti-TNF-α treatment	C	8.4 (2.9)
3.	Rituximab: administration of pneumococcal or influenza vaccination is recommended prior to treatment with this medicine (whenever possible)	C	9.8 (0.6)
4.	Rituximab: if there are indications for tetanus prophylaxis in contaminated wound management in children treated with this medicine in the past 6 months, it is suggested to administer specific tetanus immunoglobulin as the response to TT vaccine may be impaired	D	9.6 (0.9)
5.	Methotrexate: it is recommended to determine the concentration of pneumococcal strain-specific antibodies after vaccination	C	7.9 (3.4)

* Level of agreement among experts on a scale from 0 (absolute disapproval of the proposed recommendation) to 10 (total support for the recommendation). ^1^ high doses of GCs are ≥2 mg/kg BW/24 h or ≥20 mg/24 h administered over ≥2 weeks. In patients on long-term therapy, a dose of 20 mg/24 h or <2 mg/kg BW/24 h is also considered a high dose. BW: Body Weight; GCs: glucocorticosteroids; DMARDs: disease-modifying antirheumatic drugs; TNF-α: tumor necrosis factor; NIP: National Immunization Program, TT: tetanus vaccine

**Table 2:** Recommendations of EULAR expert group for administration of attenuated vaccines in children with JIA.

Recommendation Number	Treatment Regimen and Recommendation	Grade of Recommendation	Delphi Score Mean (SD)
1.	DMARDs/GCs (high dose ^1,2^), biologicals: in specific cases, administration of such a vaccine may be considered if the risk of developing an infectious disease exceeds the real risk of inducing infection by vaccination	D	9.2 (0.9)
2.	DMARDs/ GKs (GCs) (high dose ^2^): MMR and VZV booster vaccines and a YFV booster (only as a pre-exposure vaccination in travel medicine) may be considered in patients on methotrexate at a dose of <15 mg/m^2^ BSA */week or on a low-dose GCs	C	8.9 (1.5)
3.	Immunosuppressive drugs (high dose ^1,2^), biologicals: assess the patient’s VZV infection and vaccination history. In case of a negative history of VZV or vaccination, the VZV vaccine should be considered, preferably before the start of immunosuppressive therapy ^3^	D	9.2 (1.2)

^1^ High doses of GCs are ≥2 mg/kg BW/24 h or ≥20 mg/24 h administered over ≥2 weeks. In patients on long-term therapy, a dose of 20 mg/24 h or <2 mg/kg BW/24 h is also considered a high dose. ^2^ high doses of DMARDs are defined as intravenous pulse therapy of cyclosporin >2.5 mg/kg BW/24 h; sulfasalazine >40 mg/kg BW/24 h or 2 g/24 h; azathioprine >3 mg/kg BW; cyclophosphamide orally >2.0 mg/kg BW/24 h; leflunomide >0.5 mg/kg BW/24 h; 6–mercaptopurine >1.5 mg/kg BW/24 h. ^3^ It is recommended to wait at least 2–4 weeks before immunosuppressive therapy is administered. * BSA: Body Surface Area. * MMR: combined measles, mumps and rubella vaccine; VZV: varicella vaccine; YFV: yellow fever vaccine

**Table 3:** Recommendations of EULAR expert group for administration of inactivated vaccines in children with JIA.

Recommendation Number	Treatment Regimen and Recommendation	Grade of Recommendation	Delphi Score Mean (SD)
1.	Tetanus vaccination can be administered according to the local NIP.	B	9.8 (0.6)
2.	It is recommended to adhere to the local NIP for vaccinations against diphtheria, tetanus, pertussis, Hib, HBV, pneumococci and meningococci	C	9.8 (0.6)
3.	The following vaccinations can be administered in compliance with the local NIP: poliomyelitis, hepatitis A, rabies (pre-exposure vaccination in travel medicine and post-exposure vaccination), tick-borne encephalitis (pre-exposure vaccination in exposed children) and vaccination against cholera and typhoid (pre-exposure vaccination in travel medicine).	D	8.9 (1.8)
4.	Annual influenza vaccination should be considered	D	8.4 (2.2)
5.	If vaccinations against Hib, pneumococci and meningococci are not included in the current NIP, it is recommended to administer these vaccinations in patients with low complement component levels or functional asplenia. These vaccinations may also be considered in patients before the start of the treatment with high-dose immunosuppressive drugs or biologicals	D	9.3 (1.6)

**Table 4:** Vaccination procedure in children with JIA - specific tasks for nurses.

Task	Purpose
Checking the patient’s identity.	To prevent mistakes in the implementation of vaccination.
Verification of the type of vaccination to be administered against the patient’s/parent’s/guardian’s expectations and the doctor’s referral.	To identify and confront the expectations of the patient and/or parent/guardian with the indications for vaccination specified in the NIP and the recommendations of the doctor who qualified the JIA patient for the vaccination.
Verification of the written recommendation issued by the doctor who qualified the patient with JIA for vaccination.	To ensure that that there are no contraindications present and that the vaccination is administered during remission of the underlying disease.
Taking the patient’s history–adverse events and/or exacerbation of the underlying disease after previously administered vaccinations.	To reduce the risk of recurrence of the adverse event or/and exacerbation of the underlying disease.
Taking the history of vaccinations administered in the week preceding the vaccination to people in the immediate environment of a JIA patient.	To identify a potential risk of transmission of the virus contained in an attenuated vaccine, which has been administered to a household member to a patient with JIA.
Suggesting a postponement of immunization, in cooperation with the doctor who referred the child for immunization, if a person from the immediate environment of a child with JIA has recently received an attenuated vaccine.	To schedule a new date for administration of the vaccine and to inform the patient and/or parent/guardian of the need to keep the rules of personal hygiene and limit contact with the vaccinated household member for a week.
Choosing the anatomical site and the injection technique which are most suitable for a child with JIA and are in compliance with the manufacturer’s recommendations (in the case of vaccines administered via intramuscular, subcutaneous or intradermal route).	To reduce the risk of adverse events and to obtain the proper immunoprophylactic result.
Recommending the most effective method of soothing the pain that may accompany the vaccination.	To ensure the patient’s comfort after the procedure.
Administering the vaccination using the appropriate route and technique.	To ensure the proper immunoprophylactic result.
Careful documentation of the vaccination.	To identify the vaccine preparation responsible for adverse events, should they occur.
Post-vaccination observation of the child in a medical facility for at least 30 min.	To allow early identification of potential adverse events.
Providing patients with information based on reliable scientific facts regarding the need to adhere to the immunization schedule contained in the NIP for people who live with a child with JIA, who is often immunosuppressed as a result of the therapy used.	To reduce the risk of exposure of children with JIA to infectious diseases.

## Abbreviations

BCG	tuberculosis vaccine
DMARDs	disease-modifying antirheumatic drugs
GCs	glucocorticosteroids
HBV	hepatitis B vaccine
Hib	*Hemophilus influenzae* type B vaccine
HPV	human papillomavirus
JIA	juvenile idiopathic arthritis
MMR	combined measles, mumps and rubella vaccine
VAE	vaccine adverse events
PVC	pneumococcal conjugate vaccine
PPSV	polysaccharide pneumococcal vaccine (23-valent)
NIP	National Immunization Program
TNF	tumor necrosis factor
TT	tetanus vaccine
